# Influence of Green Human Resource Management on firm’s environmental performance: Green Employee Empowerment as a mediating factor

**DOI:** 10.1371/journal.pone.0293957

**Published:** 2024-04-17

**Authors:** Philip Adu Sarfo, Jianhua Zhang, George Nyantakyi, Francis Ako Lassey, Emmanuel Bruce, Ophelia Amankwah

**Affiliations:** 1 School of Management, Zhengzhou University, Zhengzhou, China; 2 School of Accounting, Zhongnan University of Economics and Law, Wuhan, China; 3 Depart of Economics, Kwame Nkrumah University of Science and Technology, Kumasi, Ghana; 4 School of Management, University of Electronic Science and Technology, Chengdu, China; Nanjing University of Science and Technology, CHINA

## Abstract

This research aimed to investigate the mediating function of Green Employee Empowerment (GEE) in the relationship between Green Human Resource Management practices (GHRM) and the environmental performance of small and medium-sized enterprises (SMEs) in Ghana, drawing on the Ability-Motivation-Opportunity (AMO) theory. This study assessed the hypotheses in the established research model using structural equation modeling based on data collected from 320 participants from small and medium-sized firms in Ghana. The study’s results revealed that GHRM practices were significantly correlated with the firm’s environmental performance. The study found significant GHRM’s indirect consequences on environmental performance through GEE in all models examined. These findings suggest that GEE plays a crucial role in translating the impact of GHRM practices into improved environmental performance. The study overlooked other potential mediators or moderators in the relationship between GHRM practices and environmental performance, focusing on GEE. To better understand the complex dynamics behind GHRM techniques’ environmental performance, future research might examine business culture, leadership style, and employee sustainability attitudes.

## Introduction

Reserving the natural environment has been a major issue for decades. Most businesses practice environmental protection. Most SMEs have increased performance by eliminating waste from the production of goods and disposal [1]. Small and medium-sized enterprises (SMEs) green production activities include decreasing waste, conserving energy and water, and educating consumers and employees [2, 3]. Environmental issues become global and create social and business challenges [4, 5]. Such has placed significant pressure on corporations to comply with environmental regulations. Production consumption patterns have several ecological, social, and economic impacts [6]. Industrial waste creation is global and resource-intensive. The use of fossil fuels in the industry is responsible for around 78% of the greenhouse gas emissions between 1970 and 2011 [7, 8]. The effects of both global warming and pollution are getting worse [9]. This research seeks to provide valuable insights into the mechanisms through which organizations can effectively enhance their environmental performance. This knowledge can be instrumental in shaping sustainable business strategies and fostering a more eco-conscious corporate culture. The study stresses green employee empowerment as a mediator. It examines how GHRM policies empower employees to promote environmental sustainability in their companies. This part of the study is essential since it highlights firms’ human environmental responsibilities.

Business groups have focused on their responsibility to environmental obligations, including climate change, pollution, and resource consumption [10]. As a result, cutting-edge approaches to environmental protection and economic growth exist. Environmental Management Systems (EMS) aid businesses and other organizations in maintaining compliance with environmental regulations through eco-friendly practices and products [11]. GHRM is the best practice for organizations adopting EMS [12].

GHRM supports its business preservation and environmental practices [13]. The policy promotes a culture within the organization that motivates employees to prioritize environmental sustainability and cost-effectiveness [12, 14]. Policies and procedures help implement the GHRM to improve HR processes [15]. Training and growth in leadership, selecting, evaluating performance, hiring, and rewards systems improve workers’ greener capabilities, encourage workers to maintain environmental sustainability, and offer green opportunities [16].

Environmental performance (EP) is a business’s attempt to exceed society’s environmental standards [17]. It concerns corporate production methods’ ecological impact and resource utilization to meet regulatory requirements [18]. The efficiency of the environment is linked to green product quality, innovation, and corporate sustainability [19]. The ability to regulate pollution, decrease waste discharge, embrace recycling and reusing methods, and deploy systems like ISO 14001 are further indicators of an organization’s environmental performance and commitment. Many environmental projects and systems demand HRM’s support. Whenever a business’s environmentally friendly ideals and HRM practices meet, EP is guaranteed [20].

Recently, businesses have been under intense pressure from stakeholders to implement corporate environmental practices. Hence, it is crucial to find sustainable green methods. Several HRM studies evaluated how GHRM affects a firm’s environmental performance [21, 22]. Again, most researchers evaluated single-variable GHRM correlations [23, 24]. Scholars have used "general" rather than just one variable to study the causal connection between HRM approaches and business performance [23, 25]. Additionally, "general" indicate an accumulation of interrelated and reliable human resource operations that complement each other [26]. GHRM research has focused on specific procedures rather than business performance [25]. According to some literature, green employee empowerment (GEE) is essential among firms to execute green tasks [27]. Empowering employees boosts productivity and engagement [28]. Muogbo [29] believed that emancipated employees feel inseparable motivation, improving work performance and satisfaction. Thus, GEE assists GHRM practices in achieving the EP.

Recent research, as exemplified by Nasir, Asad [30], has provided valuable empirical support for the hypotheses regarding the impact of Green Human Resource Management (GHRM) practices on employee pro-environmental behavior. Their findings demonstrate that GHRM practices significantly influence employees’ pro-environmental behavior, and the crucial factor of green commitment mediates this relationship. This empirical validation reinforces the relevance of the proposed hypotheses and underscores the pivotal role that GHRM practices play in shaping environmentally responsible conduct within organizations. Nasir, Asad [30] not only contributes to the growing body of literature on GHRM but also enriches the broader fields of environmental management, organizational behavior, and human resource management by highlighting the paramount importance of GHRM practices in contemporary organizational contexts.

Moreover, Green Employee Empowerment (GEE) has gained prominence in GHRM, particularly in its influence on a firm’s environmental performance. Recent studies, including those conducted by Hussain, Nazir [31, 32], and Nasir, Asad [30], have illuminated the intricate relationships among GHRM practices, GEE, and their combined impact on environmental outcomes. These studies emphasize that GHRM’s influence is multifaceted and extends beyond GEE. For instance, Hussain, Nazir [31] introduce the mediating roles of Green Transformational Leadership and Capability, highlighting GHRM’s impact’s complexity. Rehan, Abbass [32] underscore the significance of management support in fostering a culture of environmental responsibility and innovation, suggesting that GEE is part of a broader organizational ecosystem influenced by management decisions. Nasir, Asad [30] reveal that Green HRMP significantly affects employees’ commitment to environmentally friendly behavior, establishing a chain of influence from GHRM practices to employee actions. These collective insights underscore the importance of considering GEE as an integral component of the broader GHRM framework, shedding light on its potential to mediate the relationship between GHRM practices and a firm’s environmental performance. Recognizing the intricate interplay between GEE, management support, and various HR practices provides a more comprehensive understanding of how organizations can effectively promote environmental sustainability while achieving their business objectives.

GEE’s effect on the FEP-GHRM relationship has yet to be empirically explored. A considerable gap exists. Consequently, this research investigates the effects of GHRM policies on FEP across eco-friendly employee empowerment (GEE). The present study has the following contributions. First, his investigation contributes to available research by examining whether GHRM practices affect FEP in small and medium-sized enterprises and by providing actual evidence to settle the GHRMP-FEP argument since few empirical studies have been carried out in developing countries. Second, his study adds GEE to the present GHRM study by empirically investigating the process of GHRM and EP and proving the connection between GHRM practices and FEP. Third, the study contributes to the literature on GHRM by extending the mediating role of green employee empowerment and its relationship with a firm’s environmental performance. Lastly, the current study supplements the knowledge of GHRM practices in developing countries by determining its influence on the firm’s environmental performance and sustainable growth. By recognizing GEE as an integral part of the broader GHRM framework, this research offers a more comprehensive understanding of how organizations can effectively promote environmental sustainability while achieving their business objectives. The subsequent queries concerning prior studies serve as a guide for this research:

1. Does implementing GHRM practices have any bearing on the performance of the firm’s environment?
2. Does GEE mediate the relationship between the GHRM practices and FEP?

This article is structed in five parts. The first aspect introduces the study. The second part covers review of the literature, theory establishment, and study framework. The third part covers the methods. Using empirical data, the fourth aspect summarizes the investigation. The fifth part concludes the study, outlines future implications, and provides suggestions for further study.

### Review of literature

GHRM has been considered one of the most potent segments of human resource management. GRHM helps organizations enhance their human resources to boost EP and sustainability [16, 33]. GHRM practices increase employees’ green skills through recruitment, training, performance evaluation, and reward systems. GHRM strategies can sustainably shape HR efficiency, conduct, attitude, and competence [34]. Li and Sheldon [35] claim that GHRM practices reduce expenditure without harming skills, roles, or temporary employees. Meena and Girija [36] define GHRM practice as an organization’s strategy, process, and innovation to reduce environmental harm and increase positive impact.

For competitive advantage, most organizations are developing strategic EP initiatives [37, 38]. EP refers to safeguarding a business’s environment and developing measurable criteria for conducting operations within predetermined limits [25]. HR managers help achieve Environmental performance aims by employing, educating, and eco-friendly staff evaluation and incentives [21]. Researchers already emphasized HRM strategies that create staff awareness, talents, expertise, and motivations to improve the firms’ EP [39–42]

GHRM improved the utilization of resources and firm implications [43], corporate efficiency [21], public esteem and perception of the trademark (Cherian & Jacob, 2012), the firm’s contribution to the environment, and a long-term competitive edge [44]. Rawashdeh [45] recognized that businesses’ actions impact the natural world and their productivity. Much research has shown that enterprises with higher management systems for the environment profit significantly [46].

GHRM empowers employees, improving their performance and discipline. Saeed, Rasheed [21] supplemented that management of green human resources may increase employee engagement and convenience and attract talented individuals.

Recent studies, including Ali, Puah [47] and Nasir, Asad [30] have illuminated the contemporary significance of GHRM. Ali, Puah [47] highlighted the growing prominence of green-oriented organizations and their dedication to environmental sustainability. They emphasized that GHRM enhances environmental performance, stimulates innovative thinking, instills employee engagement in green initiatives, and elevates employee commitment to environmental goals.

Nasir, Asad [30] further underscored the critical role of GHRM in the present-day competitive landscape. They emphasized the challenging task HR professionals face in developing sustainable advantages for organizations in today’s era. GHRM, in this context, is seen as instrumental in heightening employee environmental awareness and elevating organizational environmental performance by promoting green behaviors. This aligns with the overarching goal of advancing environmental sustainability.

This study builds upon historical and recent insights into GHRM [39]. It seeks to investigate the impact of GHRM on a firm’s environmental performance, with a particular focus on the mediating role of green employee empowerment. By delving into this area, the research aims to contribute to the evolving discourse on GHRM and its implications for enhancing environmental performance within organizations [48].

### Contextualization of the theory

GHRM theory views GHRM practices as resources for organizations and corporate efficiency. Resource-based view (RBV) theory emphasizes how an organization’s assets, abilities, and competencies can be used to gain a competitive edge [49]. Green HRM at the employee stage is considered a strategic capability since it improves the growth of an organization [34]. GHRM develops, inspires, and provides opportunities for prospering firm behavior for the organization’s competitive advantage. If human capital uses RBV approaches to boost competitiveness, it may outperform competitors [50].

In the Ability, Motivation, Opportunities (AMO) theory, human resources strategies are based on ability, inspiration, and possibility [51]. HRM initiatives improve employee skills, incentives, and opportunities to increase business social responsibility and productivity at work [52]. Singh, Del Giudice [53] found the above hypothesis to increase productivity, reduce waste, and improve quality. Using the AMO model [54], evaluated green training, staff engagement, and hotel corporate citizenship behavior. Several AMO theory-based studies found that GHRM practices improve staff conduct and firm performance regarding the environment [51, 55, 56].

Theoretical foundations drawn from the previous studies by Ali, Puah [47], Hussain, Abbass [57], and Nasir, Asad [30] provide a solid and relevant basis for the current research objectives focused on understanding the influence of Green Human Resource Management (GHRM) practices on firm environmental performance, with a specific focus on the mediating role of Green Employee Empowerment (GEE). These studies collectively underscore the importance of integrating theories such as the Resource-Based View (RBV) theory and the Ability, Motivation, Opportunities (AMO) theory to comprehensively examine the complex dynamics between HRM practices, employee behavior, and organizational environmental outcomes.

According to Ali, Puah [47], applying the AMO theory was particularly insightful in understanding how GHRM practices could enhance academic staff’s commitment and pro-environmental behavior within a university setting. This framework aligns with the research objective of assessing the impact of GHRM practices on environmental performance. The findings in Ali, Nisar [58] study underscored the significance of providing employees with the ability, motivation, and opportunities to engage in environmentally responsible behavior. These insights are directly relevant to the current study’s evaluation of GHRM’s impact on firm environmental performance and its potential to empower employees to contribute to environmentally sustainable practices.

Hussain, Abbass [57] applied the RBV theory to explore the effects of corporate social responsibility (CSR) on environmental performance, emphasizing the role of organizational resources in influencing these outcomes. This application is relevant to the current research objectives as it highlights the importance of considering GHRM practices as valuable resources within organizations. By assessing how CSR initiatives, including green innovation and leadership, mediate the relationship between CSR and environmental performance, the study by Hussain, Abbass [57] provides valuable insights into how resources can influence environmental outcomes. These insights resonate to understand the mediating role of GEE in the relationship between GHRM practices and firm environmental performance, as it underscores the strategic importance of these practices as organizational resources.

### Green Human Resource Management practices (GHRM)

Firms benefit from GHRM implementation [59]. Researchers have employed different GHRM approaches. Green ways to handle human resources are the principal aspect of this study that aims at helping firms employ personnel with eco-friendly expertise (Recruitment and selection), assist employees in improving their skills (Training and development), evaluate employees’ everyday conduct regarding the environment (Performance Management and Appraisal), and promote conservation efforts (Reward and Compensation).

### Green Recruitment & Selection (GRS)

GHRM emphasizes hiring environmentally conscious workers to boost Environmental Performance [60] Yong, Yusliza [61] state that sustainable recruiting and selection place environmental value at the organization’s center. Green recruitment and selection attract and choose people with environmental initiatives in mind [62]. Numerous businesses now include environmental issues/actions in descriptions of duties [63]. High-level graduates prioritize their businesses’ environmental performance and credibility [42]. An environmentally conscious employer enhances a business’s identity and credibility and recruits environmentally friendly employees [64].

Recruitment approaches that ensure potential employees comprehend and approve of the organization’s green culture [28] and their environmental awareness, values, and beliefs through interviews promote green management [16]. The business’s EP is usually used for recruitment [65]. Arulrajah, Opatha [34] recommended environmental considerations in recruiting information.

### Green Training and Development (GTD)

Training and development that is environmentally friendly programs help workers overcome environmental concerns [40]. GHRM study suggests it is a crucial human or organizational trait [21]. Environmental training may increase staff awareness [34]. Employee training can improve environmental understanding, expertise, and abilities [66]. Along with educational initiatives, all firm employees should receive green training.

Green training can educate workers about eco-friendly workplace practices. Green training programs will help personnel comprehend the conservation of the environment and managing the environment and prevention, such as waste gathering and source of pollutants identification, according to [67]. A survey showed that Chinese employees’ environmental initiatives are driven by their knowledge of the environment and standards. Green knowledge management can train personnel to preserve the environment and become problem-solvers [66]. Employee engagement in eco-friendly initiatives helps set greener goals, benefits, and skills [60].

### Green Performance Appraisal (GPA)

Performance Appraisal (PA) assesses departmental EP standards and collects relevant managerial EP information [27]. According to Yusoff, Nejati [68], evaluation of performance helps personnel grow as they advance by analyzing, measuring, and comparing assumptions and achievements. Performance management strategies guarantee EP performance by fostering staff commitment to green management effectiveness [40].

A road map, scorecard, and accurate system for assessment are needed to make the workforce sustainable. Ahmad, Ullah [69] believe work descriptions should reflect green tasks and aims. According to Epstein and Roy [70], adding environmental performance within PM processes can protect the environment by harming it. Companies have learned that preventive, environmentally friendly policies and sustainable data systems for essential viable data collecting are simpler to put up [11]. Stability is critical to the business’s endeavors to achieve sustainable practices and align practice and labor force with sustainable control and success objectives [25].

### Green Compensation Management (GCM)

Management of remuneration and rewards that are environmentally friendly use monetary incentives (such as pay raises), insignificant incentives, or communal praises to drive personnel to build green competence and succeed in environmental activities. GHRM’s various environmentally friendly metrics now include managing environmental compensation [71]. Alcaraz, Susaeta [72] propose applying green compensation techniques for top leadership and all staff. The recognition and pay program attract, retains, and empowers the best employees to learn novel abilities and help the business attain its goals. Environmental management may thrive when the system for paying employees discourages offenses and promotes eco-responsiveness. Ahmad, Ullah [69] note that current firms offer incentives to staff for environmental activities. Ahmad, Ullah [69] report that 8% of UK organizations support environmentally friendly conduct with rewards and funds, suggesting this can motivate staff to build green initiatives. As previously established, incentives encourage green business practices for people and businesses.

### The mediation role of Green Employee Empowerment (GEE)

The mediating variable affects the connection between the independent and dependent variables. The present research examines green employee empowerment as a mediator between green HRM processes and environmental performance. The study also offers evidence of its impact on dependent and independent variables.

Sustainable Employee Empowerment can be seen as a prominent GHRM approach for eco-friendly corporate objectives [27]. Worker empowerment encourages participation and decision-making. It prioritizes trust, inspiration, making decisions, and reducing impediments among employees and management [73]. GHRM approaches helps to encourage employees by enhancing abilities, expertise, and performance regarding environmental reward. Muogbo [29] found that empowered personnel are internally driven, which increases job satisfaction. AMO theory proposes that GHRM practices impact employee capability and enthusiasm to attain environmentally friendly targets [51]. Norton and others stressed that worker empowerment could result in individuals performing acts that exceed the organization’s goals [74]. Tariq, Jan [27] noted that empowered greener staff are more productive, committed, and satisfied. Employee empowerment (EE) has been shown to improve organizational performance, including satisfaction with work, morale, consumer devotion and protection, and environmental sustainability. Studies show that companies with dedicated employees grow better than the general average [75, 76].

Green Employee Empowerment (GEE) has emerged as a crucial concept in the context of Green Human Resource Management (GHRM) and its impact on a firm’s environmental performance. Recent studies, including those by Hussain, Nazir [31], Tariq, Abbass [77], and Nasir, Asad [30], have shed light on the intricate relationship between GHRM practices, GEE, and their collective influence on environmental outcomes. The contemporary research by Hussain, Nazir [31] introduces the mediating roles of Green Transformational Leadership and Green Capability, highlighting the multifaceted nature of GHRM’s impact. This study emphasizes that Green Transformational Leadership and Green Capability mediate between Corporate Social Responsibility (CSR) initiatives and environmental performance. This finding underlines the complexity of GHRM’s influence and suggests that GEE is just one facet of this intricate relationship.

Tariq, Abbass [77] bring attention to the pivotal role of management support in innovation implementation, especially in the environmental context. This research emphasizes the significance of top management’s involvement in fostering an organization’s environmental responsibility culture. It further highlights how management decisions can catalyze employee involvement, knowledge management, empowerment, and aligning organizational rewards and incentives with environmental objectives. This broader perspective suggests that GEE, while important, is part of a larger corporate ecosystem influenced by management support and innovation.

Nasir, Asad [30] contribute to the discourse by exploring the link between Green HRMP (Human Resource Management Practices) and employees’ commitment to environmental goals. Their research reveals that Green HRMP, including Green Training and Development, Green Reward and Compensation, and Green Employee Empowerment, significantly influence employees’ commitment to environmentally friendly behavior. This commitment, in turn, is linked to pro-environmental behaviors, thereby establishing a chain of influence from GHRM practices to employee actions.

In this context, it becomes evident that GEE is a pivotal component of the broader GHRM framework. It is a mediating factor and an outcome of various HR practices. Green Training and Development, Green Reward and Compensation, and Green Employee Empowerment, as highlighted by Nasir, Asad [30] play essential roles in fostering employees’ commitment to environmental goals.

It is imperative to position the study within this broader landscape of recent and historical research findings to enhance its originality and scholarly impact. This comprehensive approach underscores the importance of considering GEE as part of the larger GHRM paradigm, with its potential to mediate the relationship between GHRM practices and a firm’s environmental performance. Researchers and practitioners can better understand how organizations can promote environmental sustainability by recognizing the interplay between GEE, management support, and various HR practices.

### Firm’s Environmental Performance (FEP)

Because environmental issues are becoming increasingly significant in business strategies and environmentally friendly ideas through innovations and beneficial opinions, environmental performance is seen as a mutually beneficial opportunity for increasing productivity [78]. As the public expectation for environmental sustainability rises, more businesses are using strategic procedures for environmental management (EMS) to enhance their ability to compete and incorporate environmental performance into their business plans [79]. Firms’ Environmental performance strategies reduce many business pollutants, carbon emissions, and unsafe and compacted waste. Based on [65], a system to manage the environment (EMS) (such as ISO 14001) improves environmental efficiency and demands close coordination between managing the environment and employee resources. An organization can execute the EP strategy with the right individual with the exact skills [80]. Thus, HR approaches must align with business objectives to create the talent, habits, and mindsets needed to achieve business objectives [72]. Staff must participate in environmental performance initiatives. Employees are more pleased and work harder at environmental-focused [72].

Historically, the business world primarily focused on financial performance to measure a firm’s success. However, as environmental concerns gained prominence, a paradigm shifted towards integrating environmental performance into assessing a firm’s overall success. Melnyk, Sroufe [1] highlighted the impact of Environmental Management Systems (EMS) on corporate and environmental performance. This marked the beginning of research into the link between environmental strategies and firm outcomes.

In the contemporary, businesses are adopting strategic environmental management procedures to align with these expectations and enhance their competitiveness (Yang et al., 2011). Hussain, Abbass [57] emphasize the significance of environmental strategies in the context of corporate performance.

Central to the relationship between environmental performance and human resources is Green Human Resource Management (GHRM). GHRM focuses on integrating environmentally friendly practices into HR processes and policies. It acknowledges that employees’ engagement and empowerment are vital for the success of environmental initiatives [58]. While the importance of FEP and GHRM is evident, there is still room for further research to explore the intricate relationships among these variables. This aims to fill this research gap.

### Hypothesis development

Based on present studies [81, 82], sustainable HRM practices may foster employee environmental competencies, improving environmental performance. Many observational investigations suggested employing GHRM practices to improve environmental performance (EP) by creating organizational environmental norms and principles [21]. Mousa and Othman [60] observed a good relationship between the GHRM, including practice, and the OEP in Palestinian medical care. Yusoff, Nejati [68] revealed a satisfactory connection between the GHRM group and EP in the nation’s hotel sector through organizational citizenship actions toward the environment. [25] found an excellent EP effect from GHRM practice overall. The following hypothesis is put to the test in GHRM’s practice on FEP:

Empowering employees increases their commitment to the business’s EM approach [83]. GHRM practices are strongly linked to employees’ environmental objectives [81]. The business’s eco-friendly goals depend on GEE. Green Employee Empowerment should be utilized strategically in the work environment to motivate individuals to rethink their careers, locate fulfilling jobs, and enhance their existing abilities [27]. Saeed, Rasheed [21] advises organizations to employ human resource management to safeguard the environment. Green training and participation in workforce initiatives will increase employee excitement for EM’s financial and social advantages. GHRM policies may lead to GEE because the direct environmental effort authorizes workers to achieve sustainable objectives. Given the above facts, the research proposes that;

Tariq, Jan [27] argue that green empowerment might encourage workers to participate in environmental enhancement initiatives like business engagement. Green actions include restricting the discharge of dirty water into surrounding rivers, training staff on dangerous chemical handling, and implementing GHRM rules that could motivate employees to be green oriented [84]. Effective green engagement patterns in Canadian organizations connect positively with worker demand [85]. Kaur [86] found in an ISR investigation involving 3600 employees from 41 businesses in the globe’s ten most fiscally wealthy nations that companies with little participation had lower operating and earnings profitability over three years than those with high engagement. Pinzone, Guerci [56] found that staff engagement in collaborative environmental development initiatives increases with environmental decision-making knowledge. Thus, Green Employee Empowerment (GEE) improves firm environmental performance (FEP).

Nasir, Asad [30] validates the hypotheses by demonstrating that Green Human Resource Management (GHRM) practices significantly impact employees’ pro-environmental behavior through the mediating factor of green commitment. This empirical support strengthens the hypotheses’ relevance and underscores GHRM’s pivotal role in shaping environmentally responsible conduct. Furthermore, Nasir, Asad [30] research enriches the broader literature on environmental management, organizational behavior, and human resource management by emphasizing the importance of GHRM practices in contemporary organizational contexts.

Workers are responsible for returning to FEP if they anticipate benefits from the business’s action. GHRM approaches, and FEP are linked with GEE. GHRM increases staff consciousness, passion, and engagement in green initiatives, empowering them to achieve green objectives [69]. Employee empowerment can encourage flexible policies regarding environmental conduct. Sustainable employee empowerment increases green program incentives and organization performance [27]. Long-term employee empowerment for staff indirectly affects environmental citizenship behavior. GHRM practice may enhance an organization’s ecological actions via GEE [87]. After examining relevant literature, these are the hypotheses the researcher has proposed.

H1: There is a significant relationship between GHRM practice and FEP.
H2: GEE is a mediator between GRS and FEP.
H3: GEE mediates the connection between GPA and FEP.
H4: GI is a mediator between GCM and FEP.
H5: GI is a mediator between GTD and FEP.

## Methods

### Sample procedure

Data were acquired from 320 Ghanaian SME employees from the catering/eateries, communication services, textiles making, shea butter production, electronics, and fashion domain of businesses. For data collection, HR directors were contacted by both email and phone. After discussing the research’s reasoning, some human resources administrators agreed and welcomed the author to obtain data. They urged the author to send them a survey link, which they then shared with their employees. Out of 450 given surveys, 320 responses were gathered using Google document questionnaires. All questionnaires were sent out from January 2023 until March 2023, and data was obtained. The reliability of the initial survey tool was assessed using Cronbach’s Alpha. Thirty senior managers tested the instrument. Cronbach’s Alpha tests revealed that the instruments were reliable enough (more than 0.7).

#### Ethical considerations

*Ethical approval*. The study sought ethical approval from the National Board for Small-Scale Industries (NBSSI) Ethical Committee board (SME-ERC/352/12/22). The study’s objectives and relevant information were duly disclosed to the authorities and participants.

*Consent to participate*. As required by the National Board for Small-Scale Industries Ghana, participants’ verbal permission was obtained before the study began. No participants were financially induced or compelled to participate in the study. The participants were informed that their involvement in the study was optional.

*Consent for publication*. The authors declare that the human research participants granted informed permission to publish their data.

### The measurement of different variables

To achieve the research objectives, this study employed 31 measurement instruments in the form of questionnaires and a demographic survey. Using confirmatory factor analysis and Cronbach’s Alpha, we evaluated the validity and reliability of the instrument. The test results have confirmed its reliability and validity. Utilizing a Likert scale extending from 1 (indicating strongly disagree) to 5 (indicating strongly agree) was a component of the research construct. Table 1 contains all item scales and their respective explanations.

**Table 1:** Measurements.

Variables	Dimension	Item	Source
Green Employee Employment (GEE)		4	[27, 88, 89]
Green Human Resource Management (GHRM)	Green Recruitment and Selection (GRS)	4	[36, 90, 91]
	Green Training and Development (GTD)	5	
	Green Performance Appraisal (GPA)	6	
	Green Compensation Management (GCM)	4	
Firms Environmental Performance		4	[92, 93].

### Common method bias

The present study uses a variety of methods to lessen the impact that the possibility of common technique bias might have on our findings [94]. To begin, the items to be measured were distributed around and assigned randomly to the various sections of the questionnaire. The questions have been presented in various ways, bringing us to our second point. Third, every facility provided two responses, which helped limit the possibility of bias caused by using a standard procedure.

### Data analysis procedures

The quantitative research used survey data and statistical analysis to examine the relations between GHRM practices, green employee empowerment, and a firm’s environmental performance.

Data gathered were extracted from the google forms website into an Excel worksheet for a complete data cleaning. The cleaned Excel data was then loaded into the SMART-PLS Software. This methodology is highly recommended in cases where the research objective is to forecast and investigate dependent variables to elucidate the most significant sum of variance. According to Roldán and Sánchez-Franco [95], PLS-SEM is considered the most effective approach for prediction purposes. It is also feasible to concurrently manage the measurement and structural models [95].

**Fig 1:**
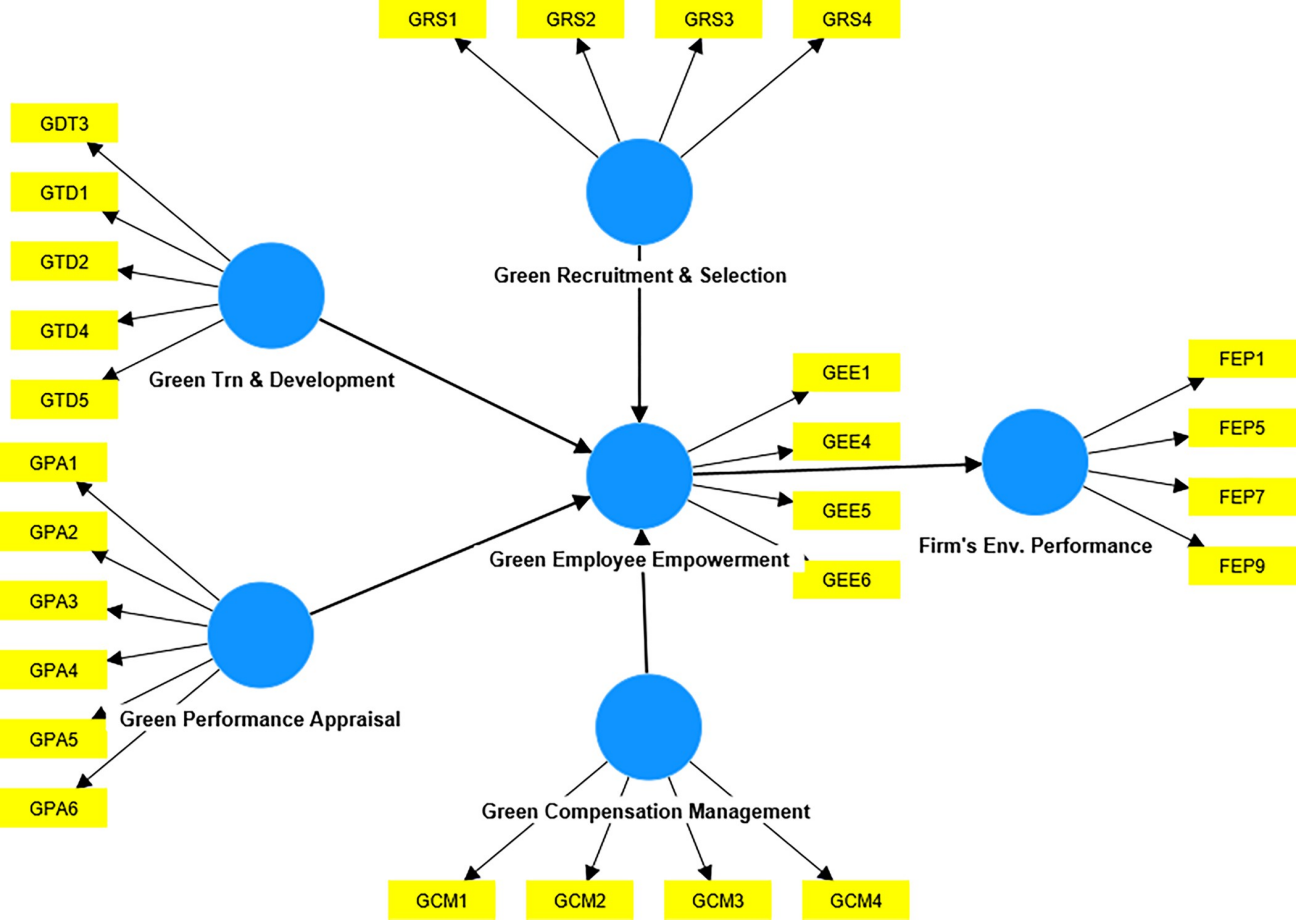
The model of the study.

Moreover, it’s an acceptable methodology for examining intricate path models. The PLS-SEM procedure can accommodate small sample sizes and yield more precise outcomes [96, 97]. Therefore, the above technique would be a suitable methodology for the present investigation. Structural equation modeling comprises a collection of statistical methods utilized to quantify and examine the associations between observable and unobservable variables [98]. This method has extensively been used in behavioral research that used primary data [99] due to its robustness. Analyzing the linear causal link between variables while considering measurement error is similar to regression analysis but more effective. Fig 1 represents the structural equation model used in this analysis.

## Results

### Respondent demographic profile

Examining the demographic characteristics of respondents is a crucial aspect of any research project. Typically, the nature and methodology employed to resolve a particular issue are influenced by the demographic characteristics of respondents. Numerous scholarly studies, such as those conducted by Othman, Latif [100, 101], contend that the demographic characteristics of survey respondents are essential for augmenting and resolving particular problems. This investigation collects demographic information from small and medium-sized business owners and employees; the results are shown in Table 2. The demographic revealed that 142 were males, representing 44.4%, and 178 were females, representing 55.6%. A bachelor’s degree was the highest qualification of the participants, representing 44.4%, and the least was a Ph.D., representing 4.7%. The position level of participants in the various organizations showed that 37.8% were general workers, the highest percentage, whereas 12.8% were top-level managers, recording the least percentage. Moreover, 30.8% have worked between 1–3 years and 29.7% for 3–5 years, the highest percentage, and very few have worked for less than a year, representing 13.8%.

**Table 2:** Profile of respondents.

Gender	**Items**	**Frequency**	**Percentage (%)**
	Male	142	44.4
	Female	178	55.6
	Total	320	100
Educational level	Bachelor Degree	142	44.4
	Diploma	74	23.1
	High School Certificate	43	13.4
	Master’s Degree	46	14.4
	Ph.D.	15	4.7
	Total	320	100.0
Position Level in Organization	General Worker	121	37.8
	Middle Manager	77	24.1
	Supervisor	81	25.3
	Top Level Manager	41	12.8
	Total	320	100.0
Years of Service	1-3years	98	30.6
	3-5years	95	29.7
	Less than a year	44	13.8
	More than 5years	83	25.9
	Total	320	100.0

### Measurement model assessment

Table 3 illustrates the results of the preliminary evaluation of the measurement data model. The validity and reliability of the data are determined through an evaluation of the measurement model using statistical techniques such as Factor Loading, Cronbach Alpha, Composite Reliability, and Average Variance Extracted (AVE). Table 3 demonstrates that each factor loading for the various constructs studied exceeded the 0.6 cutoff value recommended by [102].

**Table 3:** Evaluation of the validity and reliability of the construct.

CONSTRUCTS	ITEMS	LOADINGS (≥0.60)	Cronbach’s alpha (≥0.70)	Composite reliability (rho_c) (≥0.70)	The average variance extracted (AVE) (≥0.50)
**Firm’s Env. Performance**	FEP1	0.811	0.792	0.865	0.617
	FEP5	0.763			
	FEP7	0.774			
	FEP9	0.791			
**Green Compensation Management**	GCM1	0.803	0.796	0.868	0.621
	GCM2	0.771			
	GCM3	0.799			
	GCM4	0.777			
**Green Employee Empowerment**	GEE1	0.795	0.803	0.871	0.629
	GEE4	0.772			
	GEE5	0.815			
	GEE6	0.790			
**Green Performance Appraisal**	GPA1	0.791	0.863	0.897	0.593
	GPA2	0.742			
	GPA3	0.733			
	GPA4	0.754			
	GPA5	0.832			
	GPA6	0.766			
**Green Recruitment & Selection**	GRS1	0.774	0.785	0.861	0.608
	GRS2	0.777			
	GRS3	0.790			
	GRS4	0.778			
**Green Trn & Development**	GTD3	0.723	0.803	0.864	0.559
	GTD1	0.745			
	GTD2	0.746			
	GTD4	0.739			
	GTD5	0.786			

According to Hair, Hollingsworth [103], to determine the variables’ inner consistency, Cronbach’s alpha was more than 0.70. Composite dependability levels in the range of 0.861 to 0.897 are higher than the required value of 0.7. It is recommended that the values of the AVE should not fall below the threshold of 0.5 as a general rule [103]. Table 3 illustrates that the AVE values of the diverse constructs surpassed the threshold of 0.5, indicating a statistically favorable outcome for the analysis.

The conceptual model held 27 components for study. All items and six latent variable measures had Cronbach’s alphas and Composite reliabilities above 0.707, as shown in Table 3 [103]. The outcomes were deemed acceptable in the evaluation of recently developed measurement instruments. Based on the above, a significant level of reliability was established.

### Discriminant and convergent validities

Convergent validity is assessing coherence among various measures within a given conceptual framework. When evaluating convergent validity, it is necessary to consider the factor loading, average variance, and composite reliability derived from the indicator [103]. Interpretation of the concept may fall within a range of values from 0 to 1. According to Hair, Hollingsworth [103], the AVE value needs to exceed 0.50 to establish convergent validity. All values fall within the recommended range as outlined in Table 3.

According to [103], Fornell-Lacker specifies that diagonal elements be assigned the square roots of AVE. The square root of the AVE of the latent construct is displayed in the diagonal of **Table 4**. Furthermore, it can be deduced that the AVE increases as the row or column number increases. According to Fornell and Larcker [104] and supported by [103], the components significantly correlated with their related indicators compared to other model constructs, indicating a high level of divergent validity.

**Table 4:** Fornell-Larcker criteria.

	Firm’s Env. Performance	Green Compensation Management	Green Employee Empowerment	Green Performance Appraisal	Green Recruitment & Selection	Green Trn & Development
Firm’s Env. Performance	**0.785**					
Green Compensation Management	0.876	**0.788**				
Green Employee Empowerment	0.777	0.758	**0.793**			
Green Performance Appraisal	0.764	0.817	0.773	**0.770**		
Green Recruitment & Selection	0.739	0.693	0.709	0.757	**0.780**	
Green Trn & Development	0.743	0.737	0.733	0.785	0.773	**0.748**

### Analyzing structural models

**Fig 2:**
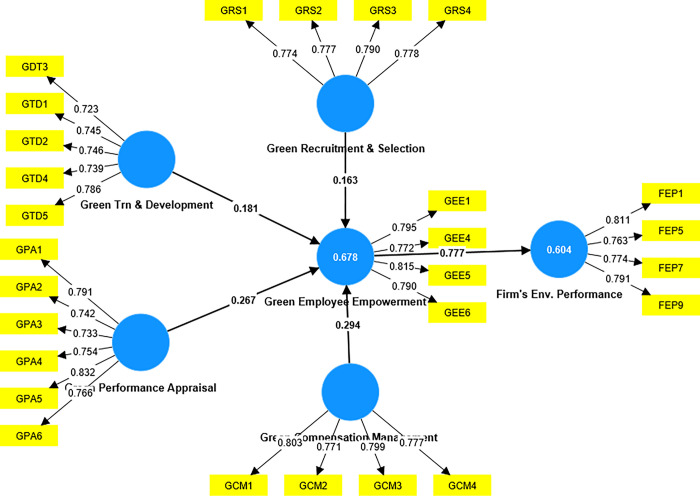
The value of the path model.

An improved version of the PLS method is utilized to analyze the structural model and investigate the interrelationships between latent variables, which are independent and dependent. The theoretical framework of the internal composition comprises a cumulative sum of six unobservable variables. The structural model’s impacts were analyzed to test the study’s assumptions, assess the model’s predictive power, and identify structures’ vitality and reliability. The four primary evaluation criteria were the path coefficient value, the T-statistic, the significance level of the model’s prediction ability (Q2), and the coefficient of determination (R2). The same procedure was used in a bootstrapping assessment [103]. The results can be seen in Fig 2.

### Coefficient and predictive relevance

The connection between the variables is substantial, moderate, or weak, and the impact is large if the R2 value is larger than 0.67, 0.33, or 0.19 [105]. This analysis showed moderate R^2^. According to Hair, Hollingsworth [103], Q^2^ values should be much higher than zero to show that the exogenous structure predicts the endogenous system. Q^2^ determines the model’s statistical importance. **Table 5** shows that FEP and GEE cross-validation values were 0.603 and 0.674, respectively. The test results were good.

**Table 5:** Coefficient and predictive relevance.

Constructs	R^2^	Q^2^
Firm’s Env. Performance	0.604	0.603
Green Employee Empowerment	0.678	0.674

### Collinearity values

Multicollinearity is a problem that can develop in many different kinds of studies depending on the nature of the research data. This problem emphasizes that exogenous variance explanations inside an endogenous structure cannot be combined to explain the variance of a single endogenous variable. The degree of multicollinearity is generally accepted using a variance inflation factor (VIF). One popular method for quantifying multicollinearity is the Variance Inflation Factor (VIF) [106]. When the most outstanding value of the VIF is greater than 5, as stated by Hair, Hollingsworth [103], multicollinearity is present. **Table 6** shows that all VIF values, comprising 1.459 to 2.266, are below five.

**Table 6:** Variance Inflation Factor (VIF).

Constructs	Items	(VIF) Outer Values	LOADINGS (≥0.60)
**Firm’s Environmental Performance (FEP)**	FEP1	1.713	0.811
	FEP5	1.501	0.763
	FEP7	1.505	0.774
	FEP9	1.604	0.791
**Green Compensation Management (GCM)**	GCM1	1.648	0.803
	GCM2	1.566	0.771
	GCM3	1.644	0.799
	GCM4	1.597	0.777
**Green Employee Empowerment (GEE)**	GEE1	1.629	0.795
	GEE4	1.544	0.772
	GEE5	1.766	0.815
	GEE6	1.653	0.790
**Green Performance Appraisal (GPA)**	GPA1	1.899	0.791
	GPA2	1.639	0.742
	GPA3	1.680	0.733
	GPA4	1.802	0.754
	GPA5	2.266	0.832
	GPA6	1.787	0.766
**Green Recruitment and Selection (GRS)**	GRS1	1.530	0.774
	GRS2	1.551	0.777
	GRS3	1.535	0.790
	GRS4	1.572	0.778
**Green Training and Development (GTD)**	GTD1	1.532	0.723
	GTD2	1.552	0.745
	GDT3	1.459	0.746
	GTD4	1.534	0.739
	GTD5	1.728	0.786

### Structural model path coefficient

Indexes and path factors determine the dependent latent variable’s internal structure model. Values for R and Q and t-value and path analyses were derived using the structural model [103].

### Examining hypotheses

Fig 2 and **Tables 7** and **8** reflect the hypothesis test procedure used in evaluating the structure of the model.

**Table 7:** Hypotheses testing.

Relationships	Path	mean (M)	Standard deviation (STDEV)	T statistics (|O/STDEV|)	P values	Decision
H1a: Green Compensation Management -> Firm’s Env. Performance	0.228	0.234	0.065	3.533	0.000	Supported
H1b: Green Performance Appraisal -> Firm’s Env. Performance	0.208	0.203	0.070	2.965	0.003	Supported
H1c: Green Recruitment & Selection -> Firm’s Env. Performance	0.127	0.124	0.050	2.543	0.011	Supported
H1d: Green Trn & Development -> Firm’s Env. Performance	0.141	0.147	0.057	2.488	0.013	Supported

**Table 8:** Evaluating indirect hypotheses.

Relationships	Path	mean (M)	Standard deviation (STDEV)	T statistics (|O/STDEV|)	P values	Decision
H2: Green Compensation Management -> Green Employee Empowerment -> Firm’s Env. Performance	0.294	0.298	0.078	3.778	0.000	Partially Meditated
H3: Green Performance Appraisal -> Green Employee Empowerment -> Firm’s Env. Performance	0.267	0.262	0.092	2.896	0.004	Partially Meditated
H4: Green Recruitment & Selection -> Green Employee Empowerment -> Firm’s Env. Performance	0.163	0.159	0.063	2.569	0.010	Partially Meditated
H5: Green Trn & Development -> Green Employee Empowerment -> Firm’s Env. Performance	0.181	0.188	0.072	2.531	0.011	Partially Meditated

This research tests four hypotheses on the connection between GHRM practices and the environmental performance of firms: green training and development, green performance appraisal, green recruitment and selection, and green compensation management. As presented in Table 8, the findings indicate that the practice of GHRM, specifically Green Recruitment & Selection, significantly correlates with the Firm’s Environmental Performance (β = 0.127, t = 2.543, p = 0.011), thereby providing support for H1c. The findings indicate that Green Training and Development predicts the Firm’s Environmental performance significantly (β = 0.141, t = 2.488, p = 0.013), thereby supporting H1d. The results indicate a significant connection among Green Performance Appraisal and Firm’s Environmental Performance, as evidenced by the beta coefficient of (0.208, t = 2.965, and p = 0.003). Therefore, hypothesis 1b is accepted. Additionally, the results (β = 0.228, t-value of 3.533, p = 0.000) suggest that Green Recruitment and Selection has a substantial connection with a firm’s Environmental Performance, thereby supporting the hypothesis of 1a. **Table 7** presents a synopsis of the findings of the hypothesis validation.

The study employed a mediation analysis to assess the mediating effect of GEE in the correlation between GHRM practices and the firm’s environmental performance, using a bootstrapping technique for estimating the indirect effect, as proposed by [107]. **Table 8** shows that Green Employee Empowerment has significantly established a link between GHRM practices and the Firm’s Environmental Performance.

The study’s outcomes illustrate that there are significant indirect effects on the Firm’s Environmental Performance through the mediation of GEE in four different models, namely H2 (Green Compensation Management -> Green Employee Empowerment -> Firm’s Env. Performance with β = 0.294, T = 3.778), H3 (Green Performance Appraisal -> Green Employee Empowerment -> Firm’s Env. Performance with β = 0.267, t = 2.896), H4 (Green Recruitment & Selection -> Green Employee Empowerment -> Firm’s Environmental Performance with β = 0.163, t = 2.569), and H5 (Green Trn & Development -> Green Employee Empowerment -> Firm’s Environmental Performance with β = 0.181 t = 2.531).

The study’s findings suggest that there is a significant statistical mediation effect of green employee empowerment (GEE) between green human resource management (GHRM) practices and a firm’s environmental performance (FEP). Therefore, the hypotheses H2, H3, H4, and H5 are all supported.

## Discussions of results

This research investigated the mediating function of Green Employee Empowerment (GEE) in the relationship between Green Human Resource Management practices and the environmental performance of small and medium-sized enterprises (SMEs) in Ghana. To address the first research question, the study tested four hypotheses about the relationship between GHRM practices and FEP. The results of the hypothesis testing revealed that GHRM practices, including Green Recruitment and selection, Green Training and Development, Green Performance Appraisal, and Green Compensation Management, were significantly correlated with the firm’s environmental performance. These findings align with previous investigation that highlights the positive impact of GHRM practices on the environment’s performance [16, 33].

The significant connections between these GHRM practices and the performance of the environment suggest that organizations can enhance their environmental performance by implementing green practices in various HRM areas. Another finding from [97] affirms that green human resource management has become a common practice for enhancing firm environmental performance. This implies that when an organization effectively practices green human resources management, it is likely that environmental performance will be improved. In addition, the outcome of this study supports the rationale behind how and why most firms have now focused on green human resource management practices to achieve environmental performance.

The study’s first finding revealed that Green Compensation Management significantly influences the Firm’s Environmental Performance *(t = 3*.*533*, *p = 0*.*000)*, supporting *H1a*. This result showed that green compensation management is pivotal for influencing and enhancing employees’ green practices. Prior studies [98, 99] have asserted that green compensation management positively contributes to a firm’s environmental performance in the hotel industry. Moreover, a study [100] also affirmed the significant role of green compensation management and a firm’s environmental sustainability performance. The second finding of the study witnessed a positive and direct effect of green performance appraisal on a firm’s environmental performance *(t = 2*.*965*,*p = 0*.*003)*, which is in line with previous studies [37, 101] that noted the significance of green performance appraisal and established its relationship with environmental performance. Given the essence of green human resource management practices in contemporary businesses, it has become imperative for firms to evaluate employees’ performance in a green environment. Thus, optimal green performance appraisal has been found to have a positive correlation with a firm’s environmental performance. This study further examined that green recruitment & selection positively and significantly affect a firm’s environmental performance *(t = 2*.*543*, *p = 0*.*011);* hence *H1c* was supported. This suggests that green recruitment and selection help to attract suitable candidates and strengthen human capital in an organization. The findings are consistent with those of [102, 103], who recommended that firms focus on potential candidates with green awareness and abilities, which ultimately affect the green environment. It was again evidenced that green training and development has a positive relationship with a firm’s environmental performance (*t = 2*.*488*, *p = 0*.*013)*, thus supporting *H1d*. This assertion has been affirmed by a recent study [104]. Further, the study highlights that green training and development leads to employee empowerment, which in turn improves their performance. Besides, green training and development help train employees and create environmental awareness towards attaining firms’ environmental sustainability goals.

In addressing the second research question, the study employed a mediation analysis to investigate the mediating role of GEE in the relationship between GHRM practices and environmental performance. The results indicated that GEE significantly mediated the association between GHRM practices and environmental performance. Specifically, the study found significant GHRM’s indirect consequences on environmental performance through GEE in all four models examined. These findings suggest that GEE plays a crucial role in translating the impact of GHRM practices into improved environmental performance. These findings align with employee empowerment as a strategic capability that enhances organizational growth and performance [34].

The study’s outcomes align with previous research by Hussain, Nazir [31], Tariq, Abbass [77], and Nasir, Asad [30], which emphasized the interconnectedness of GHRM practices, GEE and their collective influence on environmental outcomes. While GEE is a pivotal component of the GHRM framework, it is essential to recognize that it is not only a mediating factor but also an outcome of various HR practices, as highlighted by Nasir, Asad [30]. Green Training and Development, Green Reward and Compensation, and Green Employee Empowerment are crucial in fostering employees’ commitment to environmental goals.

The study’s findings contribute to existing literature by presenting compelling evidence of the positive influence of GHRM practices on environmental performance within Ghanaian SMEs. These results reinforce the perspective that adoption of GHRM policies may increase the value of a business’s environmental performance and promote sustainability, aligning with the assertions made by Dickmann and Müller-Camen [59]. Moreover, the findings emphasize the significance of empowering employees as a crucial factor in facilitating the beneficial impact of GHRM techniques on the environment’s performance. It is possible to extrapolate theoretical implications from this study by connecting the findings to applicable HRM theories. The Resource-Based View (RBV) theory emphasizes the strategic importance of GHRM practices as organizational resources that can lead to a competitive advantage. The findings of this study align with RBV theory, as they demonstrate the positive relationship between GHRM practices and environmental performance, which can contribute to the long-term competitive edge of a firm [49].

Additionally, the study draws on the Ability-Motivation-Opportunity (AMO) theory, which suggests that HRM initiatives that enhance employee skills, motivation, and opportunities can improve organizational performance [51]. The findings of this study support the AMO theory, as they indicate that GHRM practices, such as green training, performance appraisal, and compensation management, can enhance employee engagement and empower employees to contribute to environmental performance.

The study’s conclusions have theoretical significance, but they also have real-world applications. The results suggest that Ghanaian SMEs can improve their environmental performance by implementing GHRM practices, such as green recruitment and selection, green training and development, green performance appraisal, and green compensation management. These practices can help firms attract and select environmentally conscious employees, enhance employee skills and awareness of environmental issues, evaluate employee performance concerning environmental goals, and provide incentives to motivate and reward green initiatives.

Furthermore, the study illuminates the relevance of employee empowerment in mediating the connection between GHRM practices and environmental performance. Organizations should empower employees by giving them decision-making authority, fostering a culture of trust and collaboration, and offering opportunities for skill development and career growth. This can enhance employee engagement and motivation to contribute to environmental performance. This research concludes that Green Human Resource Management (GHRM) practices substantially affect Ghanaian SMEs’ environmental performance.

The research shows that environmental performance improves when GHRM practices are used, specifically Green Recruitment and selection, Green Training and development, Green Performance Appraisal, and Green Compensation Management. The investigation also shows that Green Employee Empowerment (GEE) mediates the association between GHRM practices and environmental performance, underlining the need to empower employees to achieve sustainability. These results contribute to the existing literature by providing empirical evidence of the effectiveness of GHRM practices in enhancing environmental performance. The findings have practical implications, suggesting that organizations should integrate environmental considerations into their HRM strategies to achieve their environmental goals and create a sustainable work environment.

## Conclusion

In summary, this study explores the GHRM practices’ impact on environmental performance in Ghana’s SMEs, focusing on Green Employee Empowerment (GEE) mediation. It enhances both theoretical knowledge and practical insights into GHRM and environmental performance through empirical analysis.

This study’s key findings support the proposed hypotheses, confirming a significant positive relationship between GHRM practices and a firm’s environmental performance. Specifically, Green Recruitment and selection, Green Training and Development, Green Performance Appraisal, and Green Compensation Management were linked to improved environmental performance, aligning with existing literature and highlighting GHRM’s positive impact.

Furthermore, this research underscores the pivotal role of Green Employee Empowerment (GEE) in mediating the connection between GHRM practices and environmental performance. GEE significantly mediated this relationship in various models, emphasizing the importance of empowering employees to contribute to environmental goals. These findings have theoretical significance and practical applications, suggesting that Ghanaian SMEs and organizations worldwide can enhance their environmental performance by implementing GHRM practices and fostering employee empowerment.

Despite these contributions, it’s important to acknowledge limitations. The study’s focus on Ghanaian SMEs may limit generalizability to larger organizations or diverse cultural contexts. Future research should explore additional mediating factors and moderators to advance our understanding of sustainable organizational practices.

Additionally, in alignment with the journal’s marketing and logistics focus, GHRM’s impact on environmental performance has implications for these areas. Companies adopting GHRM practices may gain a competitive edge by attracting environmentally conscious consumers. An efficient waste reduction from GHRM can positively affect supply chain management, reducing costs and improving sustainability. This aligns with the broader focus on Green Supply Chain Management (GSCM) practices, as highlighted by Tariq, Abbass [77] and Amjad, Abbas [108]. Their research highlights how GSCM practices boost organizational performance across environmental, economic, and operational dimensions. Combining GHRM and GSCM can synergize to bolster sustainability and competitive edge.

### Theoretical implications

It is possible to extrapolate theoretical implications from this investigation by connecting the findings to pertinent HRM theories. The Resource-Based View (RBV) theory emphasizes the strategic importance of GHRM practices as organizational resources that can lead to a competitive advantage. The findings of this study align with RBV theory, as they demonstrate the positive relationship between GHRM practices and environmental performance, which can contribute to the long-term competitive edge of organizations.

Again, the findings support the hypothesis that green human resource management practices (GHRM) and firms’ environmental performance (FEP) have a significant relationship. Green human resource management practices (GHRM) positively and substantially impact business environmental performance when mediated by strong green employee empowerment. This research aimed to add to the current literature on the connection between practices and firms’ performance of environment in Ghanaian SMEs. The current study augments the existing literature on the correlation between green human resource management practices and a firm’s environmental performance. Thus, the study presents a strong and comprehensive understanding of GHRM practices and the firm’s environmental performance relationship. In addition, the study delved into the mediating role of green employee empowerment and its influence on a firm’s environmental performance in the context of SMEs since limited time has been devoted to the mediating part of green employee empowerment, another significant contribution of this paper. This outcome attests partial mediating role of green employee empowerment and environmental performance in SMEs in developing countries. This implies that green employee empowerment affects employee performance and improves an organization’s environmental performance. The findings of this research may serve as a model for other developing nations to follow. Again, the study’s conclusions are essential to the academic community as they will direct scholars and other studies in the related area.

### Practical/Managerial implications

The outcome of this work has several practical and managerial inferences for businesses, particularly small and medium-sized enterprises (SMEs) in Ghana, as they seek to enhance their environmental performance and contribute to sustainable development. Thus, the current study offers a comprehensive understanding of GHRM, enabling decision-makers and business owners to recognize and acknowledge the significance of GHRM in enhancing a firm’s environmental performance. Firstly, the study highlights the importance of integrating green human resource management practices into organizational strategies. The study suggests that SME representatives can adopt GHRM practices such as environmental training programs, eco-friendly recruitment and selection processes, and performance management systems that incorporate environmental goals and targets. By doing so, organizations can create a culture of sustainability and environmental consciousness among employees, leading to improved environmental performance.

Moreover, the study findings advocate that SMEs’ environmental performance in developing economies can be improved by adopting and practicing GHRM. Furthermore, the study highlighted that GHRM requires high levels of employee empowerment. Therefore, SMEs in developing countries should provide green training and development regarding environmental protection for firm performance. Notedly, SMEs in developing nations cannot guarantee a green environment due to specific challenges, such as finances and limited resources. Knowledge about GHRM practice and environmental performance will help SMEs and regulatory stakeholders to understand these phenomena effectively and how they can be implemented to achieve and improve environmental performance.

Another contemporary practical implication is that since GHRM was found to have a positive relationship with environmental performance, SMEs, especially in Ghana and other developing countries, are recommended to adopt and implement GHRM to attract suitable candidates and enhance their sustainable performance. More importantly, the stakeholders.

### Marketing/Logistics implications

In line with this journal’s marketing and logistics focus, it’s vital to recognize the role of Green Human Resource Management (GHRM) practices in shaping environmental performance, impacting marketing and logistics significantly.

GHRM practices emphasize sustainability within the workforce, fostering eco-friendly operations that extend beyond a company’s internal functions. Businesses adopting GHRM principles and improving their environmental performance can strategically position themselves in today’s eco-conscious market. Consumers increasingly base their choices on a company’s environmental commitment, making environmental performance a potent marketing asset.

GHRM’s dedication to sustainability can attract environmentally-conscious customers, enhance brand image, and expand the customer base. For organizations, especially in marketing and logistics, GHRM practices are integral to their broader strategy, influencing market perception and interactions with eco-aware consumers. Integrating GHRM, environmental performance, and marketing creates a dynamic ecosystem where responsible practices benefit the planet and drive corporate success.

### Limitations and future research directions

This extensive study has some limitations that suggest future investigation. The study’s findings are specific to a particular region and type of business. Future studies could conduct similar studies in other countries to address this limitation. This broader geographical scope will help researchers generalize their findings and understand if the relationships they observed are consistent across different nations and cultures.

The second limitation relates to the study’s research design. The study mentions that the research employed a cross-sectional analysis. Cross-sectional studies collect data simultaneously, which can provide a snapshot of relationships but doesn’t allow for understanding how variables change over time. To address this limitation, the authors suggest using a longitudinal study design in the future. A longitudinal study collects data from the same subjects over an extended period, allowing researchers to track changes and establish causal relationships more effectively.

The third limitation is linked to the specific focus of the research on Ghanaian SMEs. It points out that because the study was conducted in this context, the findings may not be readily applicable or generalizable to other contexts or types of businesses. The authors suggest researching different industries and countries to address this limitation. This broader scope will help validate the findings and determine if the relationships between GHRM practices, green employee empowerment (GEE), and firms’ environmental performance (FEP) remain consistent in various contexts.

Moreover, the study focused on the mediating role of GEE in the relationship between GHRM practices and environmental performance, overlooking other potential mediators or moderators. In research, a mediator is a variable that helps explain the relationship between two other variables. In this case, the study looked at how certain Human Resource Management practices (GHRM) were related to environmental performance, with GEE playing a role in this relationship. While this study focused on Green Employee Empowerment (GEE) as a mediating factor, future research can explore additional mechanisms linking GHRM practices to environmental performance. For instance, investigating the role of green organizational culture, employee motivation, or innovation as mediators could provide a more comprehensive understanding of the underlying processes.

Again, investigating potential moderating variables that influence the relationship between GHRM practices and environmental performance is another promising avenue for future research. Factors such as industry type, firm size, and regulatory environment may interact with GHRM practices to affect environmental outcomes differently. Identifying these moderating variables can help organizations tailor their GHRM initiatives more effectively.

Additionally, the research relied on self-reported data, meaning the study participants provided information about themselves. This method could lead to several problems in the future. First, it could lead to "common method bias," which means that people might act in a certain way because they think that’s what is expected of them. Second, it could be subjective, which means that different people could give information based on their personal views, which could be wrong or skewed. Future studies could adopt a mixed-methods approach for data collection. This can provide a more comprehensive view of the research topic. Once more, employing objective performance metrics involves utilizing data unaffected by individuals’ viewpoints or prejudices. This could include collecting data from sources other than self-reports to make the research findings more reliable and valid.
